# Therapeutically Targeting Neuroinflammation and Microglia after Acute Ischemic Stroke

**DOI:** 10.1155/2014/297241

**Published:** 2014-06-25

**Authors:** Youngjeon Lee, Sang-Rae Lee, Sung S. Choi, Hyeon-Gu Yeo, Kyu-Tae Chang, Hong J. Lee

**Affiliations:** ^1^National Primate Research Center (NPRC), Korea Research Institute of Bioscience and Biotechnology (KRIBB), Ochang 363-883, Republic of Korea; ^2^Medical Research Institute, Chung-Ang University College of Medicine, Seoul 156-756, Republic of Korea

## Abstract

Inflammation has a pivotal role in the pathogenesis of ischemic stroke, and recent studies posit that inflammation acts as a double-edged sword, not only detrimentally augmenting secondary injury, but also potentially promoting recovery. An initial event of inflammation in ischemic stroke is the activation of microglia, leading to production of both pro- and anti-inflammatory mediators acting through multiple receptor signaling pathways. In this review, we discuss the role of microglial mediators in acute ischemic stroke and elaborate on preclinical and clinical studies focused on microglia in stroke models. Understanding how microglia can lead to both pro- and anti-inflammatory responses may be essential to implement therapeutic strategies using immunomodulatory interventions in ischemic stroke.

## 1. Introduction

Stroke is the second leading cause of death worldwide and most victims suffer from disabilities such as paresis and speech defects [1]. One of the major causes of stroke is an interruption of cerebral blood flow resulting in ischemia [2]. The incidence and mortality of stroke increase with age, and as the elderly population is rapidly growing in most developed countries ischemic stroke is a common societal burden with substantial economic costs [3]. Although great advances have been made in understanding the diverse mechanisms of neuronal cell death induced by ischemic stroke, clinically effective neuroprotective therapies are limited [1]. Recent studies suggest that cells other than neurons may be involved in the pathogenesis of ischemia and that a functional “neurovascular unit” comprises neuronal, glial, and vascular elements [4, 5].

After ischemic stroke, an inflammatory response is initiated within a few hours, with the activation of microglia and astrocytes and the production of chemoattractants, cytokines, and chemokines [6–8], with the subsequent infiltration of blood-derived cells such as leukocytes [9, 10]. These cells interact with one another via intricate signaling pathways. Recent studies show that systemic inflammatory status prior to and at the time of stroke is a key determinant of acute outcome and long-term prognosis [11, 12]. Inhibiting inflammatory responses after stroke can prevent brain injury and, therefore, improve neurological outcome [13]. Conversely, it has been suggested that suppressing inflammation could be detrimental and long-term functional recovery could be worse when inflammation after stroke is inhibited [14, 15]. Taken together, inflammatory responses after ischemic insult could be beneficial or detrimental, probably depending on the stage of stroke and environments; nevertheless, more work is needed to elucidate the role of inflammation during stroke.

Microglia are activated after ischemic stroke, changing shape and phenotype, similar to macrophages in systemic inflammation. Activated microglia have the potential to phagocytose, present antigens, and produce cytokines and matrix metalloproteinases (MMPs) that disrupt the blood brain barrier (BBB) [10]. Peripheral leukocytes can then infiltrate into the brain and further exacerbate inflammation and brain damage [15].

Interestingly, microglial activation causes the release of a number of inflammatory mediators that are either cytotoxic or cytoprotective [16]. Microglial phagocytosis contributes to restoration of tissue homeostasis by clearing pathogens and necrotic cells, suppressing inflammation, and facilitating brain repair [9, 17]. Recent studies, thus, suggest therapeutically targeting microglia in stroke. Here, we focus on the roles of microglia in neuroinflammation after ischemic stroke.

## 2. Microglia under Normal Physiological Conditions

In the resting state, microglia survey the CNS microenvironment by continuously extending and retracting ramified processes [18, 19]. They control synapse number and remodeling in the developing brain and clear debris in the healthy adult CNS [20]. Depending on the brain area, microglia can express different proteins and display various morphologies [21] and respond differently according to injuries [22]. Microglia are mostly located in the gray matter, where they ramify radial processes [21]. The activation and cell fate of microglia are influenced by their location. After focal ischemia for an hour followed by 24 h of reperfusion, the number and length of microglial processes decrease and the expression of CD11b increases in the ischemic core, indicating that microglia in this region are activated [23]. On the other hand, microglia remained inactivated with more ramified processes in the penumbra-salvageable region after reperfusion around the peri-infarct area [24]. Taken together, quiescent microglia are not simply “resting,” but rather they continuously survey and prepare to change phenotype and function in response to a variety of stimuli in their surroundings.

## 3. Microglia during Acute Ischemic Stroke

Inflammation of acute ischemic stroke is a dynamic process induced by brain-resident microglia and blood-derived leukocytes [25, 26]. Activation of microglia is the first step of the inflammatory process even within minutes [7, 27]. Two to three days following ischemia, the activation and amplification of microglia peak and continue for several weeks [28, 29]. Meanwhile, infiltration of neutrophils begins after 1 day of stroke, followed by infiltration of macrophages after 2 days of stroke [25]. Although the precise roles of microglia in ischemic stroke have not yet been fully understood, recent studies strongly suggest multiple functions. The population of microglia increased in the ipsilateral hemisphere of stroke, while it remained at basal levels in the contralateral hemisphere [30]. In the ischemic environment, microglia can phagocytose tissue debris as well as secrete proinflammatory cytokines, resulting in further damage [31]. In contrast, microglia also can secrete anti-inflammatory mediators [32, 33] to alleviate inflammation. Defective microglial activation/proliferation significantly increased the size of infarction and the number of apoptotic neurons after stroke [34], which supports the pivotal role of microglia after ischemic stroke.

### 3.1. Different Phenotypes of Activated Microglia: M1 and M2

**Figure 1:**
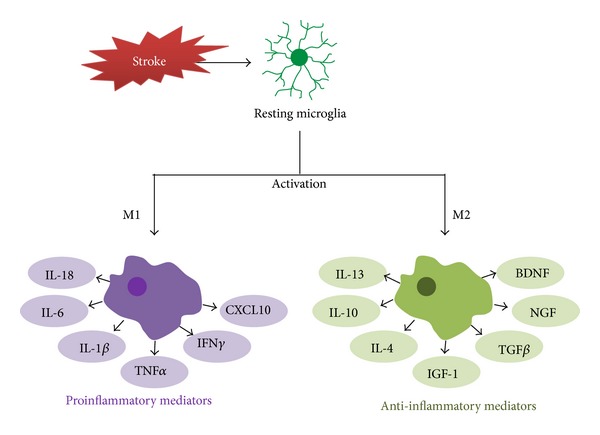
Phenotypes and activation of microglia after ischemic stroke. Under ischemic conditions, microglia change their morphology and become activated. Activated microglia are characterized as either an M1 classically activated phenotype or an M2 alternatively activated phenotype. Microglial activation induces transcription associated with the inflammatory mediators. According to their phenotypes, microglia can promote proinflammatory (by M1) or anti-inflammatory (by M2) machinery. IGF-1: insulin-like growth factor 1; IL-1*β*: interleukin-1 beta; IL-6: interleukin-6; IL-10: interleukin-10; TGF-*β*: transforming growth factor-beta; TNF-*α*: tumor necrosis factor-alpha.

During microglial activation after ischemic stroke, cell morphology is changed either to M1, the typically activated phenotype, or to M2, an alternatively activated phenotype; this phenotypic switch depends on the type of stimulation (Figure 1). M2 microglia are regarded as “healing cells” that contribute to recovery after damage and secrete anti-inflammatory mediators such as interleukin- (IL-) 10, transforming growth factor- (TGF-) *β*, IL-4, IL-13, and insulin growth factor- (IGF-) 1, as well as various neurotrophic factors [32, 35–39]. On the other hand, M1 microglia are considered as proinflammatory, producing proinflammatory meditators such as tumor necrosis factor- (TNF-) *α*, IL-1*β*, and interferon- (IFN-) *γ*. M1 microglia express CD80, CD86, and MHC class II on the cell membrane and present antigens to T cells [40]. In addition, M1 microglia tend to induce neuronal cell death more readily than M2 microglia [41]. For this reason, inhibiting the M1 phenotype has been suggested as a plausible therapeutic strategy in cerebral ischemia models. In ischemic stroke, the M2 phenotype is dominant in both local microglia and newly recruited macrophages at earlier stages, but the M1 phenotype population increases progressively in peri-infarct regions, suggesting that neurons under ischemic condition trigger changes toward the M2 phenotype in microglia and macrophages [41]. Considering these opposing roles of microglia, stroke therapies should not be focused on simply suppressing microglia but instead on balancing the beneficial and detrimental reactions of microglia.

As there is no single specific marker for microglia and activated microglia changes to amoeboid morphology with an enlarged cell body and stout processes, it is difficult to distinguish them from macrophages and myeloid-derived cells that infiltrate the injured brain tissue [42]. Since microglia and macrophages originate from primitive myeloid cells, a number of markers such as CD11b, F4/80, and Iba-1 are the same [35, 43]. Although different phenotypes of activated microglia express unique cell surface proteins, markers for each phenotype have not been determined specifically because of the similarities to other cell types. To date, several markers have been identified for activated M1 and M2 microglia (Table 1). As a marker for M1, MHC class II is used as M1 microglia participate in antigen presentation in immune reactions [44]. On the other hand, M2 microglia express high levels of antigen-presenting molecules such as Ym-1 and CD206 [24].

### 3.2. Differential Microglial Expression in Ischemic Stroke (Ischemic Core versus Penumbra)

As the number of microglia increases after ischemic stroke, the pattern of microglial response is different depending on the location of the lesion. In the ischemic core, globular Iba1^+^ED1^+^ cells appear 7 days after transient ischemia [45]. In another study, when measured 24 h after cerebral ischemia followed by reperfusion, microglia/macrophages in the ischemic core expressed high levels of CD11b, indicating activation and formation of an amoeboid phenotype [23]. Twenty-four hours after focal ischemia without reperfusion, few CD11b^+^CD68^+^ cells were identified in the ischemic core, and CD68 (same as ED1 in rodents), marker for phagocytosis, was highly expressed by day 7. At 24 h, Ym-1 and CD206, markers for the M2 phenotype, were exclusively found in the ischemic core, suggesting that the microglia/macrophages participate in tissue repair in the ischemic core [24]. Another study also confirmed M2 phenotype dominance of microglia/macrophages by finding Iba1^+^/CD206^+^ expression at 24 h after stroke in the ischemic core; the expression was highest at 5 days after insult, decreasing after 14 days [41]. As disease severity increases, however, microglia decrease in number and disintegrate; numerous dead CD11b^+^ cells were found in the ischemic core at 72 h after stroke [46], and, similarly, CD11b^+^ cells showed disintegration in the ischemic core at 7 days after permanent focal cerebral ischemia induced by photochemically induced thrombosis (PIT) method or middle cerebral artery occlusion (MCAO), whereas there are an increased number of microglia and macrophages in the penumbra [47, 48]. Altogether, it is assumed that ischemia induces injury to microglia in the infarct core in the early phase, and, subsequently, M2 microglia/macrophages migrate into the area during the first week, followed by a decrease in microglial number afterwards. In contrast, the number of M1 microglia/macrophages increases over the first 2 weeks (Table 2). Microglia respond dynamically to ischemic stroke, as an early “anti-inflammatory” M2 phenotype, followed by a transition to a “proinflammatory” M1 phenotype. Severe ischemic state of core environment including ischemic neurons could prime microglial M1 phenotype or death. These dual roles of microglia suggest that stroke therapies should be shifted from simply suppressing microglia toward adjusting the balance between beneficial and detrimental microglial responses.

Unlike the ischemic core, microglia in the penumbra seem to be highly activated [22]. In a permanent ischemic stroke model, CD68 is expressed on ramified CD11b^+^ cells in the penumbra at 6 h and, continuously, increases in the hypertrophic amoeboid cells of the ischemic core [24]. However, it should be noted that CD11b can be expressed on both resident and infiltrating phagocytes. Within 90 min of transient focal ischemia after reperfusion, the number of Iba1^+^ED1^−^ cells increases from 3.5 to 7 days and then decreases by day 14 [45]. After 8 and 24 h of focal cerebral ischemia, the length and the number of processes of microglia/macrophages in the penumbra decrease, demonstrating their activation [23]. In addition, CD11b and F4/80 are prominently expressed in the penumbra [23, 49]. Ym-1^+^ and CD206^+^ cells were not detected in the penumbra from 1 to 7 days after permanent MCAO [24], while the IBA-1^+^ and CD206^+^ cells were highest at day 5 in the penumbra after 60 min of transient MCAO [41]. The difference in animal models may account for the discrepancies between these two studies. Given that the infiltrating cells are recruited much more in permanent MCAO [50], this might be coming from a higher number of infiltrating cells or a lower survival of resident cells. Most CD68^+^ microglia/macrophages were located in the penumbra [24, 46]. Markedly proliferating resident microglia with few infiltrating blood-derived macrophages after focal cerebral ischemia were detected at 2 and 3 days of transient MCAO over 3 days after 30 or 60 min of occlusion but significantly reduced in 60 min of occlusion compared to 30 min of occlusion [7]. Moreover, in the penumbra of permanent focal ischemia, CD68^+^ microglia were accompanied by increased expression of MHC class II on the cell surface from days 3 to 7 [51]. Taken together, proliferating and activated microglia predominate in the penumbra, and their number increases over the first week after stroke. Although these studies are limited because they did not differentiate resident microglia from infiltrating macrophages, one implication is that the change of microglial phenotype occurs dynamically and consistently and the location of the microglia is crucial. This suggests that therapeutic approaches to regulate microglia need to be targeted specifically by brain region (Table 2).

In clinical studies, abundant activated microglia have been found histologically in the ischemic core as well as penumbra within 1-2 days after onset [52, 53]. Similar to the rodent stroke model, these cells remained for several weeks and were predominantly placed in the peri-infarct zone. Positron emission tomography (PET) with ^11^C-labled PK11195 enables* in vivo* imaging the presence of activated microglia [54–56]. ^11^C-labled PK11195 has significant binding potential in the core and penumbra at 2 days and remained until 30 days, however, with less specificity to differentiate inflammatory cells due to binding mitochondrial peripheral benzodiazepine receptors which are expressed in activated microglia, macrophages, astrocytes, granulocytes, and lymphocytes [57, 58]. Recently, increased [^18^F]-fluoro-2-deoxy-D-glucose (^18^F-FDG) PET imaging in the peri-infarct area shows association with activated microglia and infiltrated cells [47]. Further studies are needed to clarify the glucose metabolism and microglial response after ischemic stroke.

## 4. Therapeutic Approaches Modulating Microglial Response

### 4.1. Genes and Cells

The cytokine IL-1 has been strongly implicated in the pathogenesis of ischemic brain damage. Although ischemic damage compared with wild-type mice was not significantly altered in mice lacking either IL-1a or IL-1b alone, mice lacking both forms of IL-1 exhibited dramatically reduced ischemic infarct volumes compared with wild type (total volume: 70%; cortex: 87% reduction) [59].

Toll-like receptors (TLRs) are signaling receptors in the innate immune system that trigger specific immunological responses to systemic bacterial infection. Microglia express TLRs which lead to gene expression of proinflammatory cytokines [35]. TLR4 and TLR2 are the most marked TLRs in microglia, and they are increased after ischemia [15]. In addition, TLR4 was localized with CD11b-positive microglia in the ischemic striatum [60]. In this respect, TLR4 but not TLR3 or TLR9 knockout (KO) mice had significantly smaller infarct areas and volumes at 24 h after ischemia-reperfusion compared with wild-type mice [60]. Some previous studies focused on an important role of TLR2 signaling in brain ischemia. Temporal analysis with flow cytometry of the microglial/macrophage activation profiles in TLR2-KO mice and age-matched controls revealed reduced microglial/macrophage activation after stroke and reduced capacity of resident microglia to proliferate, as well as decreased levels of monocyte chemotactic protein-1 (MCP-1) and consequently lower levels of CD45 high/CD11b^+^ expressing cells [61].

Recently, MMPs have been regarded as important molecules in neuroinflammation as well as neuronal apoptosis. Several reports have shown that activated microglial cells are crucial in white matter lesion (WML) pathology. A transplanted microglial cell line (HMO6) and mesenchymal stem cell line (B10) migrated to sites of WMLs, including the corpus callosum (CC) and caudoputamen (CP), reduced the severity of WMLs, and inhibited the accumulation and activation of microglia and astrocytes. Transplantation of both cell types reduced the level of MMP-2 mRNA in microglia of the CC. MMP-2 protein level and activity were also both greatly reduced in the same region. These results indicate that transplantation of either microglial cells or mesenchymal stem cells could inhibit chronic cerebral ischemia-induced WML formation by decreasing MMP-2 expression in microglia and decreasing MMP-2 activity in the CC region [62].

Expressions of MMP-1, -3, -8, and -9 were significantly induced by single or combined treatment with the immunostimulants lipopolysaccharide (LPS) or phorbol myristate acetate (PMA) in primary cultured microglia and BV2 microglial cells. Inhibition of MMP-3 or -9 significantly suppressed the expression of inducible nitric oxide synthase (iNOS) and proinflammatory cytokines and the activities of nuclear factor-kappa B (NF-*κ*B), AP-1, and p38 mitogen-activated protein kinase (MAPK) in LPS-stimulated microglia [63]. Taken together, various microglia-derived cytokines, signal receptors, and neuroinflammatory proteins reported that their knockout models may play a neuroprotective role in ischemic brain injury sufficiently.

### 4.2. Chemicals

Propofol confers neuroprotection against focal ischemia by inhibiting microglia-mediated inflammatory response in a rat model of ischemic stroke. Propofol treatment reduced infarct volume and improved the neurological functions. Moreover, molecular studies showed that mRNA expression of microglial markers CD68 and Emr1 significantly increased, and mRNA and protein expressions of proinflammatory cytokines TNF-*α*, IL-1*β*, and IL-6 were augmented in the peri-infarct cortical regions of vehicle-treated rats 24 h after MCAO [64].

2,3,5,4′-Tetrahydroxystilbene-2-O-*β*-d-glucoside (TSG), an active component, has been reported to be beneficial for human health and used as an antiaging agent. Recent studies have shown that TSG presents numerous pharmacological properties including antioxidant, free radical-scavenging, anti-inflammation, and cardioprotective effects. Microglia BV2 cell lines were used to investigate the antineuroinflammatory effects of TSG. TSG reduced LPS-induced microglia-derived release of proinflammatory factors such as TNF-*α*, IL-1*β*, and nitric oxide (NO). Further, TSG attenuated LPS-induced NADPH oxidase activation and subsequent reactive oxygen species (ROS) production [65].

Cryptolepine significantly inhibited LPS-induced production of TNF-*α*, IL-6, IL-1*β*, NO, and prostaglandin E2 (PGE2). Protein and mRNA levels of cyclooxygenase-2 (COX-2) and iNOS were also attenuated by cryptolepine [66]. Kalopanaxsaponin A, a triterpenoid saponin isolated from* Kalopanax pictus*, inhibited iNOS, COX-2, and TNF-*α* expressions in LPS-stimulated microglia, while kalopanaxsaponin A increased anti-inflammatory cytokine IL-10 expression [67]. Fucoidan treatment significantly inhibited excessive production of NO and PGE2 in LPS-stimulated BV2 microglia. It also attenuated expression of iNOS, COX-2, MCP-1, and proinflammatory cytokines, including IL-1*β* and TNF-*α*. Moreover, fucoidan exhibited anti-inflammatory properties by suppressing NF-*κ*B activation and downregulating extracellular signal-regulated kinase (ERK), c-Jun N-terminal kinase (JNK), MAPK, and AKT pathways [68]. Geniposide decreased the secretion of TNF-*α*, IL-1*β*, IL-6, IL-8, and IL-10 from cultured microglial cells. It also downregulated TLR4 mRNA expression in the microglia [69]. Treatment with LXA(4) ME suppressed neutrophil infiltration and lipid peroxidation levels, inhibited the activation of microglia and astrocytes, reduced the expression of TNF-*α* and IL-1*β*, and upregulated the expression of anti-inflammatory cytokines IL-10 and TGF-*β* 1 in the ischemic brain [70]. Compared with the vehicle group, rosuvastatin prevented the impairment of neurological function and decreased the infarct volume. The increases in activated microglia, macrophages, and superoxide levels usually caused by ischemia/reperfusion were significantly ameliorated by rosuvastatin. Rosuvastatin also inhibited the upregulation of gp91^phox^ and p22phox, phosphorylation of NF-*κ*B, and induction of COX-2 and iNOS [71]. These studies suggest that chemicals such as propofol, TSG, and cryptolepine have experimentally neuroprotective effects and they may be therapeutic target for clinical application.

### 4.3. Augmentation of Anti-Inflammatory Response

Early studies showed that the administration of the anti-inflammatory cytokine IL-10 protects against permanent MCAO in mice. IL-10 was overexpressed in astrocytes, microglia, and endothelial brain cells in IL10T compared with wild-type mice. Four days following MCAO, IL-10T mice showed a 40% reduction in infarct size that was associated with significantly reduced levels of active caspase 3 compared with wild-type mice [72]. Subcutaneous administration of IGF-1 also resulted in significantly reduced infarct volumes and an increase in motor-sensory functions in normotensive rats [73].

TIPE2 (TNF-*α*-inducible protein 8-like 2 or TNFAIP8L2) is essential for maintaining immune homeostasis. Some genetic studies suggested the role of TIPE2 in the regulation of TLR function and the link between TLRs and ischemic cerebral injury. The genetic ablation of the TIPE2 gene significantly increased the cerebral volume of infarction and neurological dysfunction in mice subjected to MCAO [74].

Therefore, exogenous administration or overexpression of pivotal factors may be strong candidates for the treatment of ischemic stroke.

## 5. Conclusion

**Figure 2:**
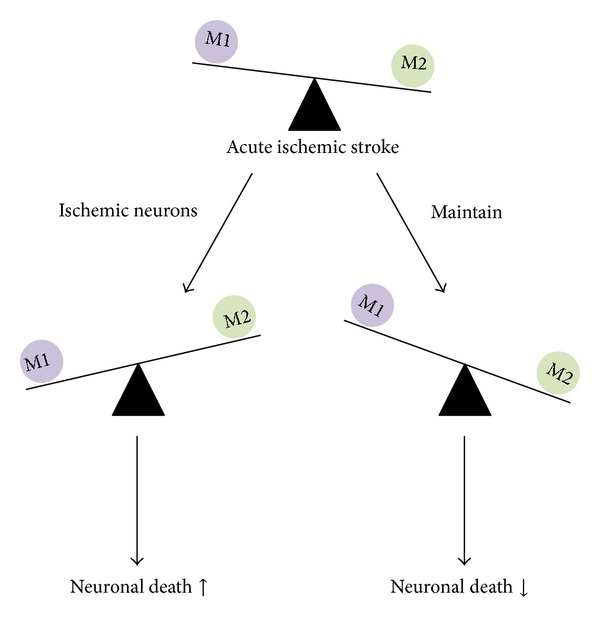
The roles of microglia in ischemic stroke. The balance between proinflammatory and anti-inflammatory responses is important for determining outcomes after stroke.

We have summarized recent evidence suggesting that microglia have critical functions during ischemic stroke. Inflammation associated with microglia plays an important role in the pathogenesis of ischemic stroke. Although several trials for anti-inflammatory treatment have proven to be effective for treating acute stroke in animal models, they have unfortunately been ineffective in clinical trials. Increasing evidence suggests that inflammatory response is a double-edged sword, as it not only exacerbates secondary brain injury in the acute stage of stroke but also contributes beneficially to brain recovery after stroke (Figure 2). Microglia could serve as powerful cellular targets in ischemic stroke. Successful microglial replacement therapy is encouraging since manipulation of microglia may be effective for treating other neurological conditions. However, there is still much to be done in order to translate promising preclinical findings into clinical practice. Further studies should consider the pro- and anti-inflammatory responses by microglia, not separately but as a whole. Improving our understanding of the dynamic balance between pro- and anti-inflammatory responses and identifying the discrepancies between preclinical studies and clinical trials may lead to the design of more effective therapies.

## Figures and Tables

**Table 1:** Markers for distinguishing activated and resting microglia.

Name	Markers	References
CD11b	Both resting and activated M/M	[24, 75]
CD45	Nucleated hematopoietic cell surface	[30, 76]
CD68 (ED1)∗	Active phagocytosis M/M	[24, 45, 77]
Iba-1	Both resting and activated M/M	[78, 79]
F4/80	Both resting and activated M/M	[35, 43]
IB4	Both resting and activated M/M	[15]
Ym-1	Activated M/M (M2)	[24]
Iba-1^+^, CD206^+^	Activated M/M (M2)	[24]
Iba-1^+^, CD16/32^+^	Activated M/M (M1)	[41]

*CD68 and ED1 are virtually the same molecule (CD68 is used more in the human context, while ED1 is the name of that protein in rodents). M/M: microglia and macrophages.

**Table 2:** Microglial marker expressions in the ischemic core and peri-infarct zone.

Location	Marker	Model	Expression (after reperfusion)	Reference
Ischemic core	Iba1	1.5 h tMCAO	24 h, 4–7 d (peak)	[45]
	Iba1^+^, ED1^+^	1.5 h tMCAO	7 d	[45]
	CD11b	1 h tMCAO	24 h	[23]
	CD68	pMCAO	7 d	[24]
	Ym-1	pMCAO	24 h	
	CD206	pMCAO	24 h	
	Iba1^+^, CD206^+^	1 h tMCAO	1 d, 5 d (peak)	[41]
	Iba1^+^, CD16/32^+^	1 h tMCAO	3 d, 14 d (peak)	

Peri-infarct zone	Iba1^+^, ED1^−^	1.5 h tMCAO	3.5 h, 7 d (peak)	[45]
	CD11b^+^, CD68^+^	pMCAO	6 h	[24]
	F4/80	pMCAO	24 h, 3 d (peak)	[49]
	CD11b	pMCAO	1 h	[23]
	CD68	pMCAO	24 h	[24]
	CD68^+^, MHC II^+^	pMCAO	3–7 d	[51]

pMCAO: permanent middle cerebral artery occlusion and tMCAO: transient middle cerebral artery occlusion.
